# Polymorphic variation of hypoxia inducible factor-1 A (*HIF1A*) gene might contribute to the development of knee osteoarthritis: a pilot study

**DOI:** 10.1186/s12891-015-0678-z

**Published:** 2015-08-21

**Authors:** Javier Fernández-Torres, Cristina Hernández-Díaz, Rolando Espinosa-Morales, Javier Camacho-Galindo, Norma del Carmen Galindo-Sevilla, Ámbar López-Macay, Yessica Zamudio-Cuevas, Karina Martínez-Flores, Mónica Guadalupe Santamaría-Olmedo, Carlos Pineda, Julio Granados, Gabriela Angélica Martínez-Nava, Marwin Gutiérrez, Alberto G. López-Reyes

**Affiliations:** Synovioanalysis Molecular Laboratory, Instituto Nacional de Rehabilitación “Luis Guillermo Ibarra”, Secretaría de Salud, Calzada Mexico-Xochimilco 289, Col. Arenal de Guadalupe, Tlalpan, 14389 Mexico, D.F. Mexico; Musculoskeletal Ultrasound Laboratory, Instituto Nacional de Rehabilitación “Luis Guillermo Ibarra”, Secretaría de Salud, Calzada Mexico-Xochimilco 289, Col. Arenal de Guadalupe, Tlalpan, 14389 Mexico, D.F. Mexico; Rheumatology Service, Instituto Nacional de Rehabilitación “Luis Guillermo Ibarra”, Secretaría de Salud, Calzada Mexico-Xochimilco 289, Col. Arenal de Guadalupe, Tlalpan, 14389 Mexico, D.F. Mexico; Escuela Superior de Medicina, Instituto Politécnico Nacional, Calle Salvador Díaz Mirón esq. Plan de San Luis, Col. Santo Tomás, 11340 México, D.F. Mexico; Cirugía Ortopédica, Centro Médico ABC, Av. Carlos Graef Fernández 154, Col. Tlaxala Santa Fe, Cuajimalpa, 05300 Mexico, D.F. Mexico; Departamento de Infectología e Inmunología Perinatal, Instituto Nacional de Perinatología “Isidro Espinosa de los Reyes”, Secretaría de Salud, Calle Montes Urales 800, Col. Lomas Virreyes, Miguel Hidalgo, 11000 México, D.F. Mexico; Escuela Nacional de Ciencias Biológicas (ENCB), Instituto Politécnico Nacional, Prolongación de Carpio y Plan de Ayala S/N, Col. Casco de Santo Tomás, Miguel Hidalgo, 11340 México, D.F. Mexico; Immunogenetics Division, Instituto Nacional de Ciencias Médicas y Nutrición “Salvador Zubirán” (INCMNSZ), Secretaría de Salud, Vasco de Quiroga 15, Col. Sección XVI, Tlalpan, 14000 Mexico, D.F. Mexico

## Abstract

**Background:**

Osteoarthritis (OA) is a multifactorial degenerative condition of the whole joint with a complex pathogenesis whose development and progression is significantly mediated by interactions between the joint cartilage and articular tissues, particularly, proinflammatory mediators and oxidative stress, which results in cartilage deterioration and subchondral bone destruction. HIF-1 alpha regulates oxygen homeostasis in hypoxic tissues such as joint cartilage; efficiency of transcriptional activity of the *HIF1A* gene is strongly influenced by the presence of polymorphic variants. Given the loss of articular cartilage and with intention to restore damaged tissue, WISP-1 participates in the development of subchondral bone; further, its expression is highly increased in chondrocytes of OA patients. The aim of this study was to evaluate gene frequencies of *HIF1A* and *WISP1* polymorphisms in Mexican patients suffering from knee OA.

**Methods:**

We determined *HIF1A* rs11549465 (P582S), rs11549467 (A588T), and rs2057482 (C191T), and *WISP1* rs2929970 (A2364G) polymorphisms in 70 Mexican patients with knee OA and compare them to those present in 66 ethnically matched healthy controls. Genotyping for these polymorphisms was performed by Real-Time PCR using TaqMan probes.

**Results:**

Gene frequencies exhibited a significant increase of the *CC* genotype of rs11549465 polymorphism in knee OA patients as compared with those present in controls (*P* = 0.003 OR = 5.7, 95 % CI = 1.7–21.6); *CT* genotype and *T* allele showed decreased frequency in the knee OA group vs. the controls (*P* = 0.003 OR = 0.2, CI = 0.05–0.6; and *P* = 0.004 OR = 0.2, CI = 0.05–0.65, respectively). Allele frequencies of the other polymorphic variants were similar in both patients and controls.

**Conclusions:**

These results suggest that the presence of the rs11549465 SNP (*HIF1A*) plays a role protective in the loss of articular cartilage in our population, and offers the possibility to further study the molecular mechanisms within cartilage and subchondral bone.

**Electronic supplementary material:**

The online version of this article (doi:10.1186/s12891-015-0678-z) contains supplementary material, which is available to authorized users.

## Background

Osteoarthritis (OA) is one of the most common rheumatic disorders; it is characterized by joint disease whose cause is not completely understood. OA has a complex pathogenesis due to the interaction of genetic, mechanical, biochemical, metabolic, endocrine, and environmental factors that result in cartilage breakdown and diverse changes in subchondral bone, which may lead to chronic pain, joint swelling, deformity and whole joint abnormalities resulting in disability [1–3]. This process entails the formation of bony outgrowths (osteophytes) and inflammation of the synovial membrane, thus triggering catabolic and proinflammatory mediators such as cytokines, prostaglandin E2, neuropeptides and nitric oxide [4–7]. Worldwide estimates indicate that 9.6 % of men and 18 % of women ≥ 60 years have symptomatic OA [8–11].

Through inflammatory mediators, biochemical injury and oxidative stress, viability of chondrocytes is compromised. Increased oxidative stress in articular cartilage has been the target of molecular research and therapeutic intervention [12–14]. Healthy articular cartilage is typically an avascular tissue, the oxygen tension ranges from 0.5 to 10 % [12]. Therefore, in order to maintain oxygen homeostasis in chondrocytes and cartilage, hypoxia-inducible factor-1 alpha (HIF-1α) is necessary to survive extremely low oxygen tensions. [12, 15, 16]. Furthermore, this transcriptional factor also has a role in the regulation of glucose transport, anaerobic energy generation and matrix synthesis by articular chondrocytes. The active site of this protein is an oxygen-dependent degradation domain (ODDD) that functions as an oxygen sensor [17, 18].

In the presence of normal oxygen tension levels, HIF-1α is hydroxylated in a proline residue within ODDD; it subsequently binds to ubiquitin and is degraded in the proteasome [17–19]. However, under hypoxic conditions, hydroxylation is inhibited and HIF-1α accumulates in the cytoplasm, is phosphorylated and translocates to the nucleus in order to activate transcription of its target genes [18–22]. Some of these target genes include nitric oxide synthase 2 (*NOS2*), vascular endothelial growth factor (*VEGF*), erythropoietin (*EPO*), some glucose transporters (*GLUT1*, *GLUT3*), Insulin-like growth factor type 2 (*IGF2*), which potentially acts in order to maintain the chondroprotective functions challenged by the detrimental conditions occurring in the OA joint environment [16, 20–25]. This relationship among different genes renders the close relation of HIF-1α with several pathologies [26–31].

It have been described polymorphic variants within the *HIF1A* gene; for example, the rs11549465 (P582S), and rs11549467(A588T) single-nucleotide polymorphisms (SNPs) are located in exon 12, on 14^th^ chromosome and influence the transcriptional activity of this gene to increase dramatically in comparison with the common isoform [32, 33].

Due to loss of articular cartilage and with the intention to restore damaged tissue, WNT inducible signaling pathway protein-1 (WISP-1) participates in the development of subchondral bone. This protein plays an important role in the formation, differentiation, growth, and maintenance of bone and articular cartilage. Active Wnt signaling contributes to osteophyte formation and might play an essential role in the anabolic pattern of joint remodeling observed in ankylosing spondylitis and OA [34]. Furthermore, it has also been showed that in both OA patients and experimental models that there is an important increase in the Wnt signaling pathway and over expression of the *WISP1* gene [35–38]. Likewise, it was discovered that variant rs2929970 (A2364G) of the *WISP1* gene (located within of the 8^th^ chromosome) is related with the development of spinal OA in postmenopausal women [39].

Because of the genetic variability of OA, the aim of this study was to analyze rs11549465, rs11549467 and rs2057482 SNPs of the *HIF1A* gene, and rs2929970 SNP of the *WISP1* gene in Mexican patients with severe knee OA.

## Patients and methods

### Study population

This study meets all criteria contained in the Declaration of Helsinki and was approved by the Ethics and Research Committee of the Instituto Nacional de Rehabilitación (INR) (Ref. INR-18/13). All participants signed an informed consent letter that included questions regarding age, gender, weight, Body mass index (BMI), and birth origin. All participants were >40 years, and declared to be native to the central region of Mexico, and to have parents and grandparents born in the same geographical region.

A total of seventy patients diagnosed with primary knee OA, and sixty-six age-matched healthy controls without symptoms or signs of OA, other types of arthritis, or any joint diseases were recruited in this study. The diagnosis of knee OA was based on the criteria of the American College of Rheumatology [40], which included primary OA with any symptoms and radiographic signs of OA according to the Kellgren-Lawrence (K&L) scale (≥2 scale). The clinical examination and radiological assessment were performed by two independent examiners who were blinded to the clinical information. The control subjects were consecutively selected among individuals without a personal and family history of OA; asymptomatic healthy subjects sex and age matched were include as control group. Other etiologies causing knee diseases such as inflammatory arthritis (rheumatoid, polyarthritic or autoimmune disease), posttraumatic or post septic arthritis, skeletal dysplasia or developmental dysplasia were also excluded.

### Sample collection

Peripheral blood samples were extracted by venipuncture from each study participant to obtain blood serum for the study of biochemical parameters, and EDTA tubes for isolating DNA.

### Determination of biochemical parameters

After collection of blood samples, the serum was separated and maintained at −80 °C until use. For determination of glucose, total cholesterol, and uric acid levels in both study groups, we employed a commercial kit (Diagnostic Systems, Germany). Quantification was performed in by spectrophotometry (iMark™, BioRad).

### Obtaining genomic DNA

Genomic DNA was extracted from 200 μL of whole blood with the commercial QIAamp® DNA Blood Mini Kit, QIAGEN (Hilden, Germany), following all of the supplier’s instructions. DNA concentration was determined by spectrophotometry (NanoDrop 2000, Thermo Scientific), and was of ~40 ng/μL.

### Selection of *HIF1A* and *WISP1* SNPs and Genotyping

Selection of SNP in *HIF1A* and *WISP1* genes was conducted based on information from the dbSNP database (http://www.ncbi.nlm.nih.gov/projects/SNP/). Three polymorphisms were included for the *HIF1A* gene and one for the *WISP1* gene in the genotyping tests (Table 1). Real-time PCR was performed in a total volume of 10 μL, that contained 40 ng DNA (1 μL), 1X TaqMan Universal PCR Master Mix (5 μL), TaqMan Probes 20X (0.5 μL), and water (3.5 μL). Thermal cycling conditions were as follows: 95 °C for 10 min, followed by 40 cycles at 92 °C for 15 s and at 60 °C for 1 min. The Rotor-Gene Q Real-time PCR System (QIAGEN, Hilden, Germany) was employed for data acquisition. Genotyping of the HIF1A and WISP1 polymorphisms were carried out using the 5′ exonuclease TaqMan Allelic Discrimination assay, which was performed utilizing minor groove binder probes fluorescently labeled with VIC or FAM and the protocol recommended by the supplier (Applied Biosystems, Foster City, CA, USA). Analysis for interpretation was performed with Rotor-Gene Q Series ver. 2.0.2 software.

**Table 1:** Single-nucleotide polymorphism (SNP) studied

Gene	Chromosome position	dbSNP rs ID	Location	SNP type
*HIF1A*	Chr14: 62207557	rs11549465	Exon 12	Pro582Ser, C1772T
	Chr14: 62207575	rs11549467	Exon 12	Ala588Thr, G1790A
	Chr14: 62213848	rs2057482	3′ UTR	C191T
*WISP1*	Chr8: 134229883	rs2929970	3′ UTR	A2364G

### Statistical analysis

The demographic and clinical data were presented as Mean ± SD and compared between groups by the Student’s *t*-test. Multivariate logistic regression was used to estimate odds ratios (ORs) and 95 % confidence intervals (CI) after adjustment for age, gender, BMI, glucose, total cholesterol, and uric acid, by using STATA ver. 12 software. The genotype and allelic frequencies were evaluated by Hardy-Weinberg equilibrium (HWE) and compared by the Chi-square test and Fisher’s exact test by using an online calculator tool (http://www.oege.org/software/hardy-weinberg.html). Allelic and gene frequencies of the *HIF1A* and *WISP1* genes from both study groups were calculated by direct count. *P* values < 0.05 were considered statistically significant. For estimating risks, we employed OR with a 95 % CI. These calculations were performed using the EpiInfo statistical program (ver. 6, Centers for Disease Control and Prevention, Atlanta, GA, USA).

## Results

The demographical variables age, gender, weight, BMI, glucose, total cholesterol, and uric acid of our study populations are depicted in Table 2. Cases were older than controls (*P* < 0.00001, 55.8 ± 6.7 vs. 47.5 ± 6.6, respectively). The mean BMI of the OA group (29.8 ± 5.1) was significantly higher than the control group (26.9 ± 4.6) (*P* = 0.0007), similar with the previous studies that reported high BMI increased the risk of developing OA. None of the individuals belonging to the control group had a BMI characteristic of obesity.

**Table 2:** Demographic, anthropometric and biochemical parameters of knee OA patients and control group

Parameters	Knee OA patients (*N* = 70)	Controls (*N* = 66)	*P* value
Male/Female (%)	11.4/88.5	10.6/89.4	0.87
Age (years)	55.8 ± 6.7	47.5 ± 6.6	**< 0.00001**
Weight (kg)	72.3 ± 14.5	68.8 ± 13.4	0.14
BMI (kg/m^2^)	29.8 ± 5.1	26.9 ± 4.6	**0.0007**
Glucose (mg/dL)	112.4 ± 43.4	102.4 ± 77.9	0.36
Total cholesterol (mg/dL)	203.0 ± 31.3	206.1 ± 60.3	0.74
Uric acid (mg/dL)	5.48 ± 1.18	6.28 ± 2.2	**0.03**

Data are expressed as means ± Standard deviation. *P* values were estimated using Student *t* test, α = 0.05. *BMI* Body-mass index = normal: 18.5–24.9; overweight: 25.0–29.9; obesity: ≥ 30.0. Normal values for glucose: 75–110 mg/dL; total cholesterol: <200 mg/dL; uric acid: 3.5–6.7 mg/dL. Significant *P* values are reported in bold

Serum levels of glucose and total cholesterol in both groups were similar in patients and controls. Mean values uric acid, were lower in the group of patients with OA as compared to those present in the control group (*P* = 0.03, 5.48 ± 1.18 vs. 6.28 ± 2.2, respectively). According to radiological KL classification, 62 patients were grade 2 and eight were grade 4.

The HWE demonstrated no differences in the distribution of all polymorphisms studied in both groups (Table 3). Table 4 shows the gene and allele frequencies of the polymorphisms studied in patients in comparison with the control group. Only rs11549465 polymorphism of the *HIF1A* gene was found to be significantly associated with increased risk of OA. The OA group exhibited an increased frequency of the *CC* genotype and the *C* allele in comparison with the control group (*P* = 0.003, 94.2 vs. 74.2 %, OR = 5.7, 95 % CI = 1.7–21.6; and *P* = 0.004, 97.0 vs. 87.1 %, OR = 5.03, 95 % CI = 1.53–18.2, respectively). For the same polymorphism, *CT* genotype and the *T* allele frequencies were higher in the control group in comparison with the knee OA group (*P* = 0.003, 25.8 vs. 5.8 %, OR = 0.2, 95 % CI = 0.05–0.60; and *P* = 0.004, 12.9 vs. 3.0 %, OR = 0.2, 95 % CI = 0.05–0.65, respectively). Distribution of rs11549467 and rs2057482 polymorphisms of the *HIF1A* gene and the rs2929970 polymorphism of the *WISP1* gene in both study groups were statistically nonsignificant.

**Table 3:** Distribution of polymorphisms in patients and controls

SNP	Genotype						HWE (*P*)	
	Patients			Controls			Patients	Controls
rs11549465	*CC*	*CT*	*TT*	*CC*	*CT*	*TT*		
	66	4	0	49	17	0	0.8056	0.2297
rs11549467	*GG*	*GA*	*AA*	*GG*	*GA*	*AA*		
	68	2	0	65	1	0	0.9034	0.9505
rs2057482	*TT*	*TC*	*CC*	*TT*	*TC*	*CC*		
	59	11	0	55	11	0	0.4755	0.4601
rs2929970	*GG*	*AG*	*AA*	*GG*	*AG*	*AA*		
	25	37	8	24	33	9	0.3018	0.6581

*HWE* Hardy-Weinberg equilibrium. If *P* < 0.05, not consistent with HWE

**Table 4:** Gene and allele frequencies of the polymorphisms studied in knee OA patients and controls

SNP	OA	Controls	*P* value*	OR	(95 % CI)
	*N* (%)	*N* (%)			
HIF1A (rs11549465)					
*CC*	66 (94.2)	49 (74.2)	**0.003**	**5.7**	1.7–21.6
*CT*	4 (5.8)	17 (25.8)	**0.003**	**0.2**	0.05–0.60
*TT*	0 (0.0)	0 (0.0)	-		
*C*	136 (97.0)	115 (87.1)	**0.004**	**5.0**	1.53–18.2
*T*	4 (3.0)	17 (12.9)	**0.004**	**0.2**	0.05–0.65
HIF1A (rs11549467)					
*GG*	68 (97.1)	65 (98.5)	1.77	0.5	0.02–76.1
*GA*	2 (2.8)	1 (1.5)	1.77	1.9	0.13–54.6
*AA*	0 (0.0)	0 (0.0)	-		
*A*	138 (98.5)	131 (99.2)	1.18	0.5	0.02–7.5
*G*	2 (1.5)	1 (0.8)	1.18	1.9	0.13–53.5
HIF1A (rs2057482)					
*TT*	59 (84.3)	55 (83.3)	2.64	1.0	0.39–2.93
*TC*	11 (15.7)	11 (16.4)	2.64	0.9	0.34–2.54
*CC*	0 (0.0)	0 (0.0)	-		
*T*	129 (92.1)	121 (91.6)	1.76	1.0	0.41–2.76
*C*	11 (7.9)	11 (8.4)	1.76	0.9	0.36–2.43
WISP1 (rs2929970)					
*GG*	25 (35.7)	24 (36.4)	2.52	0.9	0.42–2.06
*AG*	37 (52.8)	33 (50.0)	2.52	1.0	0.49–2.39
*AA*	8 (11.5)	9 (13.6)	1.98	0.7	0.24–2.58
*A*	53 (37.8)	51 (38.6)	1.78	0.9	0.58–1.63
*G*	87 (62.2)	81 (61.4)	1.78	1.0	0.62–1.74

*OA* Patients with knee osteoarthritis, *OR* Odds ratio, *CI* Confidence interval; *P value corrected by Bonferroni test, <0.05. Significant *P* values and OR are reported in bold

After adjustment of age, gender, weight, BMI, glucose, total cholesterol, and uric acid, no statistically significant associations with OA were found. Additionally, we investigate the relationship between the radiological grade of OA and the rs11549465 polymorphism showing that the grade 2 maintains a relationship with the polymorphism (*CC* genotype: *P* = 0.009, OR = 5.0; *C* allele: *P* = 0.01, OR = 4.4; *CT* genotype: *P* = 0.009, OR = 0.2; *T* allele: *P* = 0.01, OR = 0.2); whereas the grade 4 did not showed any relationship (Additional file 1).

## Discussion

As is the case of other ethnic groups, OA is the most common human arthritis in Mexicans [10], its incidence is increasing due to the current pandemic of overweight and obesity in this ethnic group. Several risk factors have been described for the development, progression and severity of OA; however, at present it is not possible to explain interindividual variability in destruction of joint cartilage. We studied Mexican patients with knee OA, some SNP in *HIF1A* and *WISP1* genes related with oxygen homeostasis in joint cartilage and bone development, and their relationship with biochemical parameters.

Our results show that the BMI in the patients with OA was higher with respect the healthy control group. This is in line with previous studies that reported how a higher BMI is a risk factor for developing knee OA, probably induced by the biomechanical joint stress [41].

Interestingly, our results showed a significant decreased level of uric acid in the OA patients with respect the healthy control. This is in line with the study of Mishra *et al*. [42], who attributed this phenomenon to the antioxidant properties of uric acid since they found an inverse correlation between uric acid and the presence of malondialdehyde (pro-oxidant agent). This induces to consider a possible link between the reduction of both the content and the antioxidant property of uric acid and the inflammatory process of OA.

Regarding the analysis of polymorphic variants of *HIF1A*, we found that the rs11549465 SNP located in the exon 12 within the *HIF1A* gene was associated in Mexican patients with knee OA. Our results show that the presence of the *CC* homozygous variant or *C* allele represent potential risk factors for development of knee OA; contrarily, we detected that the heterozygous variant of *CT* or *T* allele of the rs11549465 polymorphism of the *HIF1A* gene (in comparison with the homozygous carriers) play a protector role against the disease. This phenomenon may be explained by the fact that the presence of this polymorphism confers greater stability to the HIF-1α protein, as demonstrated by Tanimoto *et al*. [22]. In their study it has been demonstrated that the substitution of proline by serine in the 582 (P582S) position enhances its transcriptional activity due to an alteration in the characteristics and properties of the binding sites with the target genes [43]. This permit supposes a beneficial effect in maintaining cartilage homeostasis and therefore avoiding joint damage. A similar phenomenon was also obtained in other immunological disease such as type 2 *diabetes mellitus* [23].

Additionally, endochondral ossification process is triggered by the development of osteophytes. WISP-1 is an osteogenic factor and an osteoblast differentiation factor that has been studied in OA models, in which an increase in its expression was reported [32, 33]. WISP-1 is a member of the CCN family of connective tissue growth factors, which also includes WISP-2 and WISP-3. Members of the CCN family have been implicated in developmental processes such as chondrogenesis, osteogenesis, and angiogenesis. French *et al.* [44] could show that WISP-1 functions as osteogenesis potentiating factor promoting mesenchymal cell proliferation while repressing chondrocytic differentiation *in vitro*. This would suggest that the CCN family play a critical role in cartilage homeostasis. Urano *et al.* [39] analyzed the rs2929970 (A2364G) 3′UTR polymorphism in Japanese women with vertebral OA, demonstrating an increase in the *GG* genotype in the sub-group with end-plate sclerosis. This polymorphism is located in the 3′ untranslated region, within a region of splicing variation, and it may influence messenger RNA stability, translational efficiency or gene expression, and subsequently contribute to the risk of OA. Under the same context, and based on the frequency of polymorphism in our population, we decided use the same variant of the *WISP1* gene in Mexican patients with knee OA, but in our study we did not find any association. Whatsoever, it is possible that the evaluated polymorphism, do not play a major role in the development of knee OA in our population, so interesting it would be to explore other polymorphic sites within the *WISP1* gene. It will be important to replicate this study in distinct ethnic groups to better understand the mechanisms whereby gene interacts with environmental factors to develop OA.

We are aware that a larger cohort of patients is needed in order to strengthen the statistical power of results, and this aspect is the main limitation of our study. However we believe that our preliminary results could be of interest since that there are no papers focused in this topic, especially involving a population with particular genotype such as Mexican population, and could differ from that typically is reported in other populations. Further investigation involving large cohort of patients and different centers could support better these preliminary results*.*

## Conclusions

In conclusion, our results suggest that the presence of the rs11549465 SNP (*HIF1A*) plays a role protective in the loss of articular cartilage in our population, and offers the possibility to further study the molecular mechanisms within cartilage and subchondral bone. Thus, HIF-1α would be a useful molecular tool for the development of new diagnostic markers, as well as of therapeutic target in OA.

## Additional file

Additional file 1:
**Gene and allele frequencies of the rs11549465 polymorphism in OA patients according the Kellgren-Lawrence scale.** (DOC 42 kb)
