# Supplementary material for: Polymorphic variation of hypoxia inducible factor-1 A (HIF1A) gene might contribute to the development of knee osteoarthritis: a pilot study
Source: BMC Musculoskelet Disord. 2015 Aug 21;16:218. doi: 10.1186/s12891-015-0678-z (PMC4546180; doi:10.1186/s12891-015-0678-z)
Supplement: Additional file 1: — Gene and allele frequencies of the rs11549465 polymorphism in OA patients according the Kellgren-Lawrence scale. (DOC 42 kb) [file 12891_2015_678_MOESM1_ESM.doc]

**ADDITIONAL FILE 1**

**Gene and allele frequencies of the rs11549465 polymorphism in OA patients according the Kellgren-Lawrence scale.**

|  | **OA group** |  | **Control group** |  |  |  |  |
| --- | --- | --- | --- | --- | --- | --- | --- |
| **K&L Grade 2, N=62** | **n** | **F** | **n** | **F** | ***P**** | **OR** | **(95% CI)** |
| *CC* | 58 | 0.93 | 49 | 0.74 | **0.009** | **5.0** | 1.46 – 19.0 |
| *CT* | 4 | 0.06 | 17 | 0.25 | **0.009** | **0.2** | 0.05 – 0.69 |
| *TT* | 0 | 0 | 0 | 0.00 | - |  |  |
| *C* | 120 | 0.97 | 115 | 0.87 | **0.01** | **4.4** | 1.35 – 16.1 |
| *T* | 4 | 0.03 | 17 | 0.12 | **0.01** | **0.2** | 0.06 – 0.74 |
|  |  |  |  |  |  |  |  |
| **K&L Grade 4, N=8** |  |  |  |  |  |  |  |
| *CC* | 8 | 1.00 | 49 | 0.74 | NS |  |  |
| *CT* | 0 | 0 | 17 | 0.25 | NS |  |  |
| *TT* | 0 | 0 | 0 | 0.00 | NS |  |  |
| *C* | 16 | 1.00 | 115 | 0.87 | NS |  |  |
| *T* | 0 | 0 | 17 | 0.12 | NS |  |  |

#### OA, Patients with knee osteoarthritis; K&L, Kellgren and Lawrence; OR, Odds ratio; CI, Confidence interval; F, gene and allele frequencies; *P value corrected by Bonferroni test, <0.05; NS, not significant; Significant P values and OR are reported in bold.
